# Differential spontaneous recovery across cognitive abilities during detoxification period in alcohol-dependence

**DOI:** 10.1371/journal.pone.0176638

**Published:** 2017-08-02

**Authors:** Géraldine Petit, Olivier Luminet, Mariana Cordovil de Sousa Uva, Alexis Zorbas, Pierre Maurage, Philippe de Timary

**Affiliations:** 1 Research Institute for Psychological Sciences, Université catholique de Louvain, Louvain-la-Neuve, Belgium; 2 Department of Adult Psychiatry, Saint-Luc Academic Hospital, Université catholique de Louvain, Brussels, Belgium; 3 Laboratory for Experimental Psychopathology, Psychological Sciences Research Institute, Université catholique de Louvain, Louvain-la-Neuve, Belgium; 4 Institute of Neuroscience, Université catholique de Louvain, Louvain-la-Neuve, Belgium; 5 The Belgian National Fund for Scientific Research (FRS-FNRS), Brussels, Belgium; 6 Unité Intégrée d’Hépatologie, Saint-Luc Academic Hospital, Université catholique de Louvain, Brussels, Belgium; Monash University, AUSTRALIA

## Abstract

**Objective:**

There is a lack of consensus regarding the extent to which cognitive dysfunctions may recover upon cessation of alcohol intake by alcohol-dependents (AD), and the divergent findings are most likely due to methodological differences between the various studies. The present study was aimed at conducting a very strict longitudinal study of cognitive recovery in terms of assessment points, the duration of abstinence, control of age and duration of the addiction, and by use of individual analyses in addition to mean group comparisons. Our study further focused on the 2–3 week phase of alcohol detoxification that is already known to positively affect many biological, emotional, motivational, as well as neural variables, followed by longer-term therapies for which good cognitive functioning is needed.

**Methods:**

41 AD inpatients undergoing a detoxification program, and 41 matched controls, were evaluated twice in terms of five cognitive functions (i.e., short-term memory, working memory, inhibition, cognitive flexibility, and verbal fluency) within a three-week interval [on the first day (T1) and the 18^th^ day (T2) of abstinence for AD patients]. Emotional (positive and negative affectivity and depression) and motivational (craving) variables were also measured at both evaluation times.

**Results:**

Although verbal fluency, short-term memory, and cognitive flexibility did not appear to be affected, the patients exhibited impaired inhibition and working memory at T1. While no recovery of inhibition was found to occur, the average working memory performance of the patients was comparable to that of the controls at T2. Improvements in emotional and motivational dimensions were also observed, although they did not correlate with the ones in working memory. Individual analysis showed that not all participants were impaired or recover the same functions.

**Conclusions:**

While inhibition deficits appear to persist after 18 days of detoxification, deficits in working memory, which is a central component of cognition, are greatly reduced after alcohol detoxification. Individual differences in the trajectory of recovery do arise however, and it might be worth implementing individual assessments of impaired functions at the end of the detoxification phase in order to maximize the chances of success in longer-term treatments and abstinence.

## Introduction

It has long been known that chronic alcohol abuse leads to a wide-range of mild to moderate cognitive impairments; mostly of executive, memory, and visuospatial functions [1]. The extent to which these dysfunctions may recover with cessation of alcohol intake in alcohol-dependent (AD) subjects has been investigated extensively over the past several decades. As these investigations have often been based on cross-sectional comparisons between groups of AD patients with different durations of abstinence (e.g., [2]), they do not provide entirely reliable information regarding recovery. Several studies have used longitudinal designs to directly monitor within-individual changes over time (see [3] for a review). Despite this approach, findings regarding many functions such as memory, working memory, and other executive functions have remained very inconsistent, as both recovery and persistent deficits with sustained abstinence have been reported for the same cognitive function. For example, Fujiwara et al. [4] saw no improvement in working memory over time in detoxified patients, while Manning et al. [5], and Sullivan et al. [6] found the opposite to be the case. Unlike Loeber et al. [7] and Fujiwara et al. [4], Durazzo et al. [8] and Rosenbloom et al. [9] found that enhancement of verbal short-term memory abilities occurred in line with the duration of abstinence. In contrast to Manning et al. [5], several research teams (e.g., Fujiwara et al., [4], Durazzo et al., [8], and Cordovil de Sousa Uva, [10]) saw no improvement in inhibition skills with abstinence. Inconsistencies in temporal assessment time points, varying periods of abstinence prior to the first testing session (i.e., the baseline), and a lack of consideration of determining factors on cognitive recovery such as age or the duration of the addiction may underlie these contradictory results. Indeed, the brains of younger individuals have been shown to be more vulnerable to alcohol-related insult [11], while they are also thought to be able to recover or compensate for damage better than the brains of older individuals [12]. The extent of the cognitive deficits are however proportional to the length of the dependence [13]. Moreover, many studies did not control for practice effects by testing the control participants more than once. Lastly, in these studies the recovered/non-recovered status for cognitive functions was based on group average performances. It is however very likely that these average values comprise individual differences, and that not all participants follow the same recovery trajectory.

The aim of the present study was to overcome these limitations by conducting a very strict longitudinal study of cognitive recovery in which all of the participants were tested after the same time of abstinence prior to the first cognitive testing, and then retested after the exact same time period. The controls were also tested twice with the same time interval as the patients, in order to distinguish the effect of practice from that of spontaneous recovery. Age and the duration of addiction were also included as covariates in the analyses, and specific attention was paid to individual performances.

Moreover, this study focused on a specific time period, i.e., the alcohol detoxification phase. This phase, that usually lasts two to three weeks, indeed constitutes a crucial initial treatment stage in AD treatment, and it is already known to be accompanied by improvement in many AD associated symptoms. Biological parameters, such as the levels of cortisol [14], leptin [15], intestinal permeability [16,17], lipopolysaccharides [16], or systemic inflammation [18] improve rapidly within this short-term abstinence period. Emotional and motivational states also improve rapidly, with less depressive-anxious symptoms and diminished alcohol craving [10,14,19–21]. Clinical observations from our alcohol unit, in which patients are hospitalized for two weeks with a one week interval during which they return home, have shown that abstinence also rapidly provides AD individuals with a better control of alcohol consumption. In most cases (70%) they are able to return home without relapsing, while it was entirely impossible for them to stop drinking based on their own initiative when they were admitted to the unit. The patients frequently report feeling more confident in regard to their ability to manage high-risk situations that may result in resumed consumption. This subjective sense of increased control may well be due to rapid recovery of the cognitive functions associated with controlled behaviors. The fact that inflammation, cortisol levels, and emotional state, which all improve within the 2–3 weeks of detoxification, have also been found to be related to cognition functioning [22–27] supports this idea. Lastly, a recent MRI-based study [28] has shown significant recovery of grey matter volume in several brain regions after only two weeks of abstinence. These anatomical changes may hence parallel very rapid and concomitant recovery of some cognitive functions.

Acquiring detailed knowledge regarding the potential short-term cognitive recovery during the specific period of the inpatient detoxification phase may have important clinical implications for treatment strategies. The 2–3 weeks of detoxification in a hospital are often followed by post-treatment programs requiring efficient cognitive functioning [29] in order to maintain long-term motivation to change drinking behavior, as well as to learn, retain, and apply strategies provided by a therapist so as to avoid relapse. These range from making new healthy lifestyle choices to dealing with situations that used to trigger alcohol consumption, by first recognizing them and secondly by resisting the urge to repeat old automatic behaviors, as well as by instead setting up a more adaptive behavioral pattern, putting an end to any drinking situation that may still be encountered before it becomes a full-blown relapse, dealing with the negative emotional feelings in case of failure [30], etc. All of these abilities rely heavily on verbal and visual learning and memory, as well as executive functioning. Impaired cognitive abilities during a therapeutic program are thus likely to reduce the efficacy of this intervention and the possibility of abstinence following detoxification, as confirmed by the link between cognitive deficits in early abstinence and relapse rates [31]). Obtaining a precise description of the differential pattern of recovery across cognitive functions at the end of the detoxification thus constitutes a crucial research aim.

Yet little is known to date regarding the recovery of cognitive abilities at the early stages of abstinence (i.e., during the first 2–3 weeks of detoxification). Most studies have explored the potential cognitive recovery in medium-term (i.e., several weeks [5,32–37]) or long-term (i.e., more than one year [9, 38, 39] abstinence. Only one study of our group [10] investigated cognitive recovery in a very short time frame; i.e., after 14 to 18 days of detoxification. We found that deficits in cognitive flexibility, selective attention, decision making, and inhibition observed at the beginning of the detoxification were still present when it ended.

The aim of this study was to further explore the potential short-term recovery of cognitive functions during detoxification. We assessed five different cognitive functions: the three main executive functions of Miyake [40], i.e., flexibility, working memory updating (WM) and inhibition of prepotent response. We also assessed verbal short-term memory (STM) and verbal fluency for which there is still a lack of consensus in regard to their change after detoxification (see [3] for a review) and which were not investigated in the previous study using a similar design [10]. We investigated changes in these cognitive functions during detoxification in AD patients, using a test—retest design at the beginning (day 1) and end (day 18) of detoxification. Emotional and motivational dimensions such as craving, affectivity, and depressive symptoms, which are known to evolve with early detoxification, were also evaluated. Given the suggested link between emotion and cognition deficits in AD (e.g., [27,41]) correlations between potential improvement in cognition and improvement in emotion and/or motivation (craving) were explored. Following what was found in the previous study [10], we made the hypothesis that inhibition and flexibility would be lower in AD compared to controls and would not improve by T2. No firm hypothesis was made concerning the three other functions given the inconsistent previous findings.

## Materials and method

### Participants

Forty-one AD patients and 41 healthy controls, matched for gender and educational level, were tested. All of the AD participants were selected based on a psychiatric interview according to DSM-IV criteria, and they were recruited during a detoxification program at the Department of Adult Psychiatry of the Saint-Luc Academic Hospital and in the alcohol dependence ward of the Clinique La Ramée (Brussels). AD patients were diagnosed as alcohol-dependent using the DSM-IV criteria [42]. Upon initiation of the treatment, the patients were administered benzodiazepine medication (diazepam: 28.7±14.1 mg/day at T1) that was progressively tapered (10.3±15.2 mg/day at T2). Patients who did not consume alcohol at the start of the detoxification, who relapsed during their stay, who presented addictive comorbidities (e.g., dependence on drugs other than alcohol (except nicotine) based on the DSM-IV criteria [42]), or symptoms of dementia were excluded. The control participants were recruited by word of mouth, and they were paid for their participation in the study. They were all free of current or past DSM-IV psychiatric disorders on Axes I or II, and they were not taking any psychotropic medications. All of the controls consumed less than 21 standard drinks/week (14 drinks for women) or 3 drinks/day (2 drinks for women). They did not report any history of addiction. The current study was approved by the ethics committees of the hospitals (i.e., Comité d'Ethique Hospitalo-Facultaire, Cliniques Universitaires Saint Luc, Brussels, Belgium. Comité d’éthique La Ramée Fond Roy Sanatia, Parhélie, Brussels, Belgium, and all of the participants signed an informed consent form.

### Procedure

The patients were tested twice within a three week interval (on the first day (T1) and the 18^th^ day (T2) of abstinence). The controls were also tested twice, with the same time interval as the patients, in order to separate practice effects from spontaneous recovery. Each time, all of the participants were tested in french with the exact same procedure: after describing the study and obtaining informed consent, demographic data were obtained, participants completed the questionnaires assessing motivational and affective states, and they were tested on several cognitive tests.

### Questionnaires: Affective-motivational variables

#### The Obsessive-Compulsive Drinking Scale (OCDS)

It measures the cognitive aspects of alcohol craving in the previous weeks [43–45]. OCDS is a 14-item self-report questionnaire (Total = Tot), divided into two subscales: a 6-item ‘obsessive’ subscale (Ob) and an 8-item ‘compulsive’ subscale (Co). Four compulsion items, related to actual alcohol consumption, were removed as the patients were abstinent, thus leading to a modified 4-item compulsive subscore (Com) and a modified 10-item total score (Totm).

#### The Positive Affectivity Negative Affectivity Schedule (PANAS)

It is a 20-item scale assessing positive and negative mood states [46,47]. It consists of 10 positive (PA) and 10 negative (NA) adjectives rated on a 5-point scale.

#### The Beck Depression Inventory (BDI)

It is a self-report 21-item inventory measuring the severity of depressive symptoms [38].

### Cognitive tasks

#### The WM task

We used a modified and computerized version [48] of the *Brown—Peterson task* [49,50]. This task evaluates the retrieval of 24 items after an interference task. First of all, three consonants (a trigram) are presented on the screen. Next, in the memory task, subjects have to hold the trigram in their working memory for a delay period, varying from 0 to 20 seconds (i.e., 0, 5, 10, or 20 seconds) while performing an interfering task (e.g., verbally presented pairs of numbers are repeated backwards). Lastly, immediately after the delay, a signal notifies the subjects that their response is due. They are required to verbally recall the three consonants in the order that they were previously presented to them. The percentage of correct recalls for each delay period is recorded.

#### The cognitive flexibility task

We used the *Number-Letter task* [51]. This test includes three subtasks. In the non-executive “Number” task (NT), twenty numbers are printed in red. The participant is required to indicate the numerical position of each number in the sequence from 1 to 9 as BEGINNING (if 1 to 3) or END (if 7 to 9). In the second non-executive “Letter” task (LT), twenty letters printed in blue are presented. The participant has to indicate the position of each letter in the alphabet: BEGINNING (if A, B or C) or END (if X, Y or Z). In the executive functioning “Number-Letter” task (N/LT), the 20 items displayed consist of pairs composed of a number and a letter. The pairs are alternately printed in either blue or red. When the pair is red, the participant has to provide the accurate response (‘beginning’ or ‘end’) based on the numerical position of the number. When the pair is blue, they need to provide the alphabetical position of the letter. The number of errors and the mean reaction times (RTs) (global time/number of items) are recorded for each subtask. Additionally, a switching index (SI) is calculated using the following formula:

$$\text{mean RTs “Number}/\text{Letter ” condition}-\;\frac{\left(\text{mean RTs “Number” condition }+\text{ mean RTs “Letter” condition}\right)}{2}$$

#### The verbal fluency task

We used the Word Fluency Tests, in which participants have to quote as many words as possible from a category in a given period of time (e.g., 60 seconds). Words beginning either with the letter R or V are requested in the Phonetic Word Fluency test, and animals or fruits are requested in the Semantic Word Fluency test. The number of correct words quoted is recorded.

#### The inhibition of prepotent response task

We used a computerized version [52,53] of the *Stroop color naming task* [54]. The stimuli are written in either blue or red. For each experimental session, the stimuli are presented individually on the screen for a maximal duration of 10 seconds. Participants are instructed to respond to the color of each stimulus as fast as possible and to accurately press the key congruent with the color (e.g., X for blue vs. N for red) in which the words are written, while attempting to ignore the meaning of the word itself. In the *interfering condition* (IC), words expressing color and the colors in which they are displayed are incongruent. In the *facilitating condition* (FC), the two stimulus dimensions are congruent. A *neutral condition* (NC) with percentage marks is also included for comparisons. Medians (mRTs) rather than means for each condition are calculated (for correct responses only) in order to reduce the influence of extreme RTs.

Additionally, an interference index (II) is calculated from these mRTs as follows:

$$\frac{\text{mRTs incongruent items }–\text{ mRTs neutral items }\times 100}{\text{mRTs neutral items}}$$

The interference index has the advantage of removing the psychomotor component, as it only takes into account the executive component. The interference score is therefore a good index of inhibition deficit.

#### The STM task

We used the Digit Span task [55,56]. This is a simple verbal test, which requires the storage and the rehearsal of sequences of digits right after they are presented verbally. In this auditory test, the experimenter says numbers slowly, in one-second intervals, in a monotone voice. The person has to respond by repeating the sequences of numbers in the correct order. If the sequence of the numbers is remembered correctly, the experimenter adds a digit to the next sequence until the participant fails. The number of correctly remembered digits is recorded.

### Statistical analyses

For demographic variables, between-group differences were tested using independent t- and chi-square tests. For motivational, affective, and cognitive measures, a logarithmic transformation was computed for variables for which the distribution did not meet the normality (i.e., the Stroop and the Brown-Peterson tests). Secondly, 2 (Group) X 2 (Time) ANOVAs with repeated measurements were conducted with Time (T1 vs. T2) as within-subjects factor and Group (AD vs. C) as between-subjects factor on experimental measurements. For cognitive dimensions, age and the duration of the addiction were added as a covariates in ANOVAs (thus becoming ANCOVAs), as they are known to influence cognitive recovery [57]. The observed interactions were supplemented by Bonferroni post-hoc tests. Only Group main effects and Group X Time interactions were reported, as we were interested in differences between AD patients and controls and potential recovery in patients. For the tasks for which we found a group effect, performance was further computed on an individual basis for both evaluation times from a deviance criterion at a threshold of 1.65 SD from the mean of the control group (i.e., this corresponds to the fifth percentile of a normal distribution, which is a common threshold to highlight deviance from the mean (e.g., [58]). Lastly, we investigated correlations between improvement in emotional and motivational (craving) variables and improvement in cognition. Statistical analyses were performed using IBM SPSS Statistics for Windows software (Version 22.0, IBM Corp., Armonk, NY).

## Results

### Descriptive statistics

The average duration of the addiction for the patients was 9.41 (7.82) years. No difference was found between groups in terms of their level of education [t(163.079) = 1.213, p = 0.227] or gender [χ2(1) = 1.952, p = 0.162], although the mean age of the patients was higher [t(79.965) = 2.264, p = 0.026]. Demographic values are presented in Table 1.

**Table 1:** Sociodemographic data for the clinical and the control groups.

Variable	AD N = 41	Control N = 41	Significance
Mean age, ± SD	49.54 ± 11.58	43.80 ± 11.34	p = 0.026^a^
Gender, N (%)			
Male	30 (73.2)	24 (58.5)	NS^b^
Female	11 (26.8)	17 (41.5)	NS^b^
Mean years of addiction, ± SD	9.41 ± 7.82	/	/
Mean educational level* (years), ± SD	14.85 ± 3.57	14.51 ± 3.14	NS^a^

NS = p <0.05

Abbreviations: AD = alcohol-dependent, SD = standard deviation, NS = not significant, N = number of participants.

*Number of years of education since completing primary school

^a^ Independent sample t-tests

^b^ Chi-square tests

### Craving

Our analyses revealed a main effect for Time [*F*(1.70) = 69.414, *p*<0.001, η^2^ = 0.498; decrease of craving with time], Group [*F*(1.70) = 189.845, *p*<0.001, η^2^ = 0.731; higher craving in the patients], and a Group by Time interaction [*F* (1.70) = 60.609, *p*≤0.001, η^2^ = 0.464; significant decrease in craving over time exclusively for the patients] for the total craving. The same effects and interactions were found for the obsessive [Time: *F*(1.70) = 48.599, *p*<0.001, η² = 0.410; Group: *F*(1.69) = 170.619, *p*<0.001, η^2^ = 0.709; Time X Group: *F*(1.70) = 49.429, *p*<0.001, η^2^ = 0.414], and compulsive [Time: *F*(1.69) = 71.455, *p*<0.001, η^2^ = 0.509; Group: *F*(1.69) = 150.722, *p*<0.001, η^2^ = 0.686; and Time X Group: *F*(1.70) = 51.523, *p*<0.001, η^2^ = 0.427] subcomponents.

### Affective states

#### Positive affects

A Group X Time interaction was found, *F*(1.80) = 5.64, *p* = 0.02, η^2^ = 0.066, explained by an increase of PA with time exclusively for the patients.

#### Negative affects

Our analyses revealed main effects for Time [*F*(1.80) = 6.531, *p* = 0.012 η^2^ = 0.075; decrease of NA with time], and Group [*F*(1.80) = 32.883, *p*<0.001, η^2^ = 0.291; higher NA in the patients].

#### Depression

Our analyses revealed a main effect for Time [*F*(1.76) = 25.953, *p*<0.001, η^2^ = 0.255; decrease in depression with time], Group [*F*(1.76) = 59.677, *p*<0.001, η^2^ = 0.440; higher depression in the patients], and a Group X Time interaction [*F*(1.76) = 20.935, *p*<0.001, η^2^ = 0.216; decrease in depression with time only in the patients]. Motivational and emotional values are presented in Table 2.

**Table 2:** Motivational and emotional measures in alcohol-dependent patients vs. control participants.

	AD patients		Control participants		Main effects		Interaction
Tests and Parameters	Time 1 AD (N)	Time 2 AD (N)	Time 1 C (N)	Time 2 C (N)	Time T1vs. T2	Group AD vs. C	Time x Group
OCDS, mean ± SD							
Obsession	10.87 ± 5.31 (32)	4.97 ± 3.22 (32)	2 ± 1.11 (40)	0.225 ± 1.27 (40)	**	**	**
Compulsion	8.52 ± 3.46 (32)	3.61 ± 2.92 (32)	0.72 ± 1.47 (40)	0.32 ± .86 (40)	**	**	**
Total	19.53 ± 8.34 (32)	8.47 ± 5.59 (32)	0.92 ± 2.05 (40)	0.55 ± 1.97 (40)	**	**	**
PANAS, mean ± SD							
PA	29.19 ± 7.46 (41)	32.17 ± 7.41 (41)	30.12 ± 7.48 (41)	29.63 ± 7.57 (41)	ns	ns	*
NA	18.41 ± 6.57 (41)	16.31 ± 4.55 (41)	12.66 ± 2.63 (41)	12.22 ± 3.15 (41)	*	**	ns
BDI, mean ± SD	20.34 ± 11.92 (38)	11.02 ± 7.62 (38)	4.15 ± 5.33 (40)	3.65 ± 5.37 (40)	**	**	**

* p < 0.05, ** p < 0.001.

Only cases (excluding missing values) are included

Abbreviations: AD = alcohol-dependent, C = controls, OCDS = Obsessive and Compulsive Drinking Scale; PANAS = Positive Affectivity Negative Affectivity Scale, PA = Positive Affect, NA = Negative Affect; BDI = Beck Depression Inventory, NS = not significant, SD = standard deviation, N = number of participants.

### Neuropsychological testing

#### The cognitive flexibility task

Given that the error rate for this task was low (i.e., <4%) and that no difference in the error rate was observed between the groups, our analysis only focused on reaction times. A main effect for Group was found for the Letter subtask [*F*(1.77) = 5.930, *p* = 0.017, η^2^ = 0.072], indicating a slower response in AD compared to controls. No effect of Group was found for the other subtasks and the switching index (SI) [NT: p = 0.060; N/LT: p = 0.187; SI: p = 0.431], nor was there a Time X Group interaction (p>0.170). Further individual analyses showed that despite the difference between the groups, only one patient was below the controls’ average at T1, and none were so at T2.

#### The verbal fluency task

No Group main effects or Time X Group interactions were observed for either the phonetic word fluency [G: p = 0.503, TXG: p = 0.350] or for the semantic word fluency [G: p = 0.828, TXG: p = 0.575].

#### The inhibition of prepotent response task

Given a floor effect of error rates (less than 6%) for all stimuli, the analyses only focused on the logarithmic transformation of mRTs for congruent, neutral, and incongruent stimuli. No Group or interaction effect was found for the facilitating [G: p = 0.124, TXG: p = 0.605] and the neutral (NC) conditions [G: p = 0.510, TXG: p = 0.127]. We found main Group effects however for the incongruent condition [*F*(1.74) = 21.435, p<0.001, η^2^ = 0.225] and the interference index [*F*(1.74) = 11.571, p = 0.001, η^2^ = 0.135], which indicates that the interfering condition reduced the patients’ performance and their processing speed. No significant Time X Group interaction (p>0.317) was found. Individual analysis showed that in the incongruent condition where a group effect was found, 56% of the patients exhibited a deviation from the controls’ mean at T1; and only 43% still exhibited an impairment, relative to this mean, at T2.

#### The STM task

No significant main effect for Group (p = 0.698) or Time X Group interaction (p = 0.123) was found.

#### The WM task

**Fig 1:**
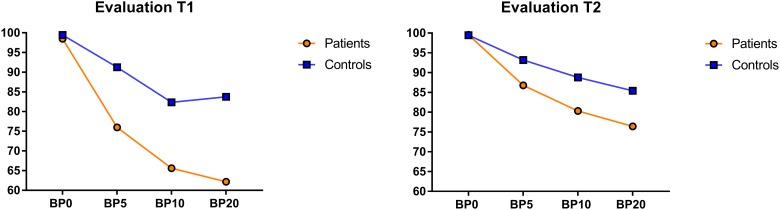
Recall percentage in the Brown-Peterson Task at T1 and T2 for the AD patients (in orange) and the Controls (in blue) for the 5, 10, and 20 second delays.

No significant Group main effect or Time X Group interaction was observed for either the immediate recall [G: p = 0.683, TXG: p = 0.894], or for the 5 second delay [G: p = 0.157, TXG: p = 0.506]. An interaction between Time and Group was found however for the 10 second [*F*(1.72) = 8.440, p = 0.005, η^2^ = 0.105] and 20 second delays [*F*(1.73) = 7.050, p = 0.010, η^2^ = 0.088]. These results firstly demonstrate that the patients, and not the controls, improved their performance between T1 and T2 [Diff T1/T2: AD: 10 seconds: p>0.001; 20 seconds: p = 0.007; C: 10 seconds: p = 0.879; 20 seconds: p = 0.831], and secondly that while the AD and the controls differed at T1 [Diff AD/C: 10 seconds: p = 0.002; 20 seconds: p>0.001], this was no longer so at T2 [Diff AD/C: 10 seconds: p = 0.664; 20 seconds: p = 0.404]. Secondly, in order to control for a potential effect of the amount of valium received at the time of each evaluation on the WM performance that could explain the improvement observed in patients, separate ANCOVAs were run for each evaluation time (T1 and T2) with the amount of valium at T1 and at T2 entered as covariates. The four (condition: 0 second delay vs. 5 second delay vs. 10 second delay vs. 20 second delay) X 2 (Group: AD vs. C) repeated measurements ANCOVA for T1 showed a main effect of Group [F(1.72) = 7.159, p = 0.009, η^2^ = 0.090], and a Condition X Group interaction [F(3.216) = 4.615, p = 0.005, η^2^ = 0.060]. Pairwise comparisons showed that while there was no difference between the patients and the controls for the 0 second and 5 second delays, the patients recalled fewer consonants than did the controls for the 10 second (p = 0.006) and 20 second delays (p = 0.010). The same analysis performed for T2 showed that there were no longer Group (p = 0.704) or Condition (p = 0.788) effects. Lastly, individual analysis showed that for the 10 second delay, 32% of the patients were not in the normal range at T1, and at T2 this was the case for 20% of the patients. For the 20 second interval, 56% of the patients were impaired at T1, while at T2 68% of the patients exhibited a performance equal to that of the controls. Detailed performances for all cognitive tasks are presented in Table 3. Fig 1 presents the performance of controls and patients for each delay in T1 and T2.

**Table 3:** Neuropsychological performances in alcohol-dependent patients vs. control participants^a^.

	AD patients		Control participants		Main effects			Interaction
Tasks	Time 1 AD (N)	Time 2 AD (N)	Time 1 C (N)	Time 2 C (N)	Time T1 vs. T2	Group AD vs. C	Age	Time x Group
**Stroop test, median RTs (ms) ± SD**								
**Congruent**	979.14 ± 526.30 (41)	823.76 ± 297.72 (39)	653.93 ± 191.28 (40)	622.53 ± 125.13 (39)	ns	**	†	ns
**Neutral**	865.50 ± 320.99 (41)	771.24 ± 245.72 (39)	713.55 ± 230.10 (40)	662.30 ± 212.70 (39)	ns	†	*	ns
**Incongruent**	1175.20 ± 499.02 (41)	1070.9 ± 551.71 (39)	728.31 ± 239.84 (40)	672.96 ± 169.19 (39)	ns	***	**	ns
**Interference Index**^b^	34.17 ± 23.03 (41)	34.81 ± 32.82 (39)	4.46 ± 21.53 (40)	3.01± 22.27 (39)	ns	***	ns	ns
**Number-Letter, mean RTs (ms) ± SD**^c^								
** Number**	18.30 ± 6.97 (40)	17.64 ± 8.46 (40)	12.74 ± 2.48 (41)	13.15 ± 3.48 (41)	ns	***	***	ns
** Letter**	18.27 ± 7.48 (40)	16.92 ± 6.69 (40)	12.20 ± 2.46 (41)	12.49 ± 4.47 (41)	ns	***	**	ns
** Number/letter**	54.87 ± 23.72 (40)	57.22 ± 22.86 (40)	42.63 ± 11.1 (41)	40.53 ± 10.56 (41)	ns	**	**	ns
** Switching index**^d^	36.59 ± 20.18 (40)	39.94 ± 17.60 (41)	30.16 ± 10.21 (40)	27.70 ± 10.25 (41)	ns	*	*	*
**Phonetic Fluency, mean (Nbr) ± SD**	17.15 ± 5.32 (39)	18.87 ± 6.46 (39)	19.98 ± 6.07 (41)	19.95 ± 5.13 (41)	ns	ns	ns	ns
**Semantic Fluency, mean (Nbr) ± SD**	25.28 ± 9.41 (39)	23.15 ± 9.31 (39)	25.73 ± 7.21 (41)	24.63 ± 7.91 (41)	ns	ns	ns	ns
**Digit Span task, mean (Nbr) ± SD**	5.56 ± 1.07 (41)	5.41 ± 1.07 (41)	5.82 ± 1.06 (41)	5.09 ± 1.09 (40)	ns	ns	ns	ns
**BP, mean (%) ± SD**								
**Interval 0 sec**	98.5 ± 4.47 (37)	99.55 ± 2.74 (37)	99.44± 2.45 (40)	99.44 ± 2.45 (40)	ns	ns	ns	ns
**Interval 5 sec**	75.97 ± 22.26 (37)	86.79 ± 19.52 (37)	91.25 ± 13.83 (40)	93.19 ± 11.83 (40)	ns	*	ns	ns
**Interval 10 sec**	65.61 ± 24.53 (37)	80.33 ± 22.85 (37)	82.36 ± 19.60 (40)	88.77 ± 15.80 (40)	ns	*	ns	†
**Interval 20 sec**	62.19 ± 23.97 (36)	76.43 ± 25.6 (36)	83.75 ± 16.85 (40)	85.41 ± 19.44 (40)	ns	**	ns	*

^a^ Only cases (excluding missing values) are included.

^b^ The Interference Index was calculated as $\frac{\text{median RTs on incongruent items }-\text{ median RTs on neutral }\times \;100}{\text{median RTs on neutral}}$

^c^ Mean time per item/total number of items

^d^ The Switching Index was calculated as $\text{mean RTs }“\text{Number}-\text{Letter }”\text{ condition }-\;\frac{\left(\text{mean RTs }“\text{Number}”\text{ condition }+\text{ mean RTs }“\text{Letter}”\text{ condition}\right)}{2}$

†0.05 < p < 0.10, * p <0.05, ** p <0.01, *** p <0.001.

Abbreviations: AD = alcohol-dependent, C = controls, SD = standard deviation, median RTs = median reaction times (milliseconds), ms = milliseconds, Nbr = number, % = percentage of successful performances, BP = Brown-Peterson, N = number of participants.

#### Correlations between improvements in WM and improvements in emotional variables and craving

The Spearman product—moment coefficient was used to test the correlations between the improvement (i.e., the difference in performance between T1 and T2) in the Brown-Peterson task and the improvement in depressive symptoms, affects, and craving in AD patients. No significant correlation was found (p>0.332).

## Discussion

Many biological, emotional, motivational, as well as neural variables are positively affected by a 2-3-week alcohol detoxification program. The main question addressed in the current study was the effect of a 2-3-week alcohol detoxification program on several cognitive functions (e.g., working memory, verbal short-term memory, verbal fluency, inhibition, and cognitive flexibility). Our study was based on a very strict design in terms of assessment points, duration of the abstinence, control for age and duration of the addiction, while it also comprised individual analysis in addition to mean group comparisons.

In the cognitive flexibility task, we only observed a difference between groups in the Letter condition, independently of the evaluation time. AD were slower, on average, when they had to determine the alphabetical position of a letter that was presented to them. The individual analysis revealed however that only one patient had a performance deemed to be significantly below the mean value for the controls. The analysis of the number/letter condition, which constitutes the actual measure of flexibility in the task, did not reveal any difference between AD and controls at either point in time. AD patients thus exhibited flexibility abilities that were equal to those of the controls upon initiation of the treatment, and they did not improve when tested 18 days later. This finding is in keeping with prior studies showing a normal ability to modify planned actions and to simultaneously maintain more than one train of thought in AD subjects who were abstinent for a year [59] as well as for shorter periods, i.e., 6 months [7] and even 6 weeks [32]. Our results provide additional information by showing that this capacity is preserved from the very beginning of detoxification. However, they contrast with other studies showing impairment in cognitive flexibility in AD patients with three weeks [60–62] or even longer periods of abstinence (e.g., up to one year) [63], and also with our hypothesis based on a previous study that found that there was impairment in flexibility and no recovery during detoxification [10].

No difference in verbal fluency was found between the groups, either at the beginning or at the end of the hospital stay. The same absence of a difference at both evaluation times was observed for the 0 second and 5 second delay conditions of the Brown-Peterson task, and for the digit span task, both of which involve only short-term storage and rehearsal of verbal-acoustic information. These results are consistent with the majority of cross-sectional studies that found no difference in verbal fluency (e.g., [61] (after 3 weeks of abstinence), [64] (after 6 weeks of abstinence), or STM capacity (e.g., [65], 6 weeks to 16 years of abstinence) between detoxified AD and controls. Our results provide additional clarification in regard to the intactness of these cognitive abilities, even in AD who continue to consume alcoholic beverages. These findings contrast however with other studies that have reported deficits in short-term memory (e.g., [9], 15 weeks of sobriety) or verbal fluency (e.g., [5], 4 days of abstinence) in detoxified AD, although a degree of recovery was also found to occur subsequently.

Our results regarding inhibition of prepotent responses confirmed our hypothesis and earlier findings [10]: AD patients exhibit impaired functions when starting treatment and no improvement occurred by the time the detoxification ended. This is also consistent with other cross-sectional studies of early abstinent AD [60,62,63,66]. The fact that the deficit was only revealed by the reaction times is in line with the suggestion that speed alone is responsible for several cognitive impairments in AD [67–69]. Lastly, while no Time X Group interaction was found for the Stroop task, individual analysis revealed that the proportion of patients that exhibited a performance below the mean of the controls was reduced by 13% from T1 to T2.

For working memory, our results reveal a lower performance in AD compared to controls at T1 for the 10 second and 20 second delays. However, some improvement was observed between T1 and T2 in AD, with their performance being found to be similar to that of the controls at T2 for both the 10 second and 20 second delays. Our finding of impaired working memory functioning at the beginning of detoxification is consistent with previous studies (e.g., [70], 3^rd^ day of detoxification). Our results are also in line with the findings of Manning et al. [5] who reported recovery in working memory from day 4 to day 26 after detoxification. However, it contradicts studies showing impairments in working memory in AD with longer abstinence periods (e.g., [65], 6 weeks to 16 years). The individual analysis further revealed that the deficits in AD for the 10 second delay actually only occurred in one-third of the patients at T1, and one-third of them reached normal performances at T2. For the 20 second delay, 56% of the patients were impaired at T1, and half of them recovered by T2.

Lastly, consistent with previous research [10,14,19–21], we observed a decrease in craving scores and depressive symptoms, and an increase of PA in AD patients between T1 and T2. As in previous studies, the magnitude of these changes in scores between the two times points were medium to large. However, no correlation was found between these improvements and the improvements observed in the WM task. It would hence appear that, unlike what has been suggested previously (e.g., [27,41]), the changes in WM abilities occur independently of those for emotion and motivation. This could however be due to the small sample size. Furthermore, it would be worth testing whether improvements in WM could be related to short-term biological changes that have been noted in previous studies focusing on the detoxification phase, in particular in terms of the level of cortisol [71] or inflammation [17]. Elevated cortisol levels have indeed been linked to neuropsychological dysfunction as a result of cell damage in the hippocampus and the frontal cortex (e.g., [72]), and several lines of evidence suggest that inflammatory cytokines are responsible for cognitive impairments [73].

The finding that WM abilities recover soon after detoxification has important implications. WM is tightly linked with other crucial abilities for addicted individuals targeting abstinence. Individuals with optimal WM also perform better in terms of reasoning and problem solving (i.e., fluid intelligence, [74,75]). Being able to efficiently reason and solve problems is crucial for patients going through a recovery process and who experience negative affects and alcohol urges that could lead to reconsumption. A high WM capacity has also been linked to a high ability of directing attention toward goal-relevant information and of ignoring distractions, i.e., attentional control [76–78]. Chronic alcohol abusers often exhibit excessive attentional focus on alcohol-related cues [79] that could increase the risk of relapse [79–81]. Effective attentional control is hence essential to overcome these attentional biases and to maintain abstinence. Lastly, individual differences in WM abilities have been linked to emotion regulation skills [38,39], for which the role in the control of alcohol usage has been documented many times [30,82]. WM is hence at the root of many other crucial abilities that are needed for post-detoxification treatments (e.g., cognitive behavioral therapy, motivational enhancement, and support groups) to be successful, and its integrity at the end of the detoxification process is therefore of considerable importance. Our individual analyses however showed that there is variability among patients. Not all of the patients exhibited impairments at T1, and not all of the impaired patients recovered by T2. Given the importance of WM, clinicians should be attentive to patients who might not quickly regain these WM abilities. In light of this, it might be worth engaging in systematic individual evaluations at the end of the detoxification stay, and to provide rehabilitation (e.g., [83]) for those in need.

Several conclusions may be drawn from our results. Firstly, our data showed differential patterns in the cognitive functions that were investigated, and these depended on the trajectory of recovery during the detoxification: inhibition was impaired at both times (with quite sizeable effects, thus indicating pronounced deficits), verbal fluency, cognitive flexibility and STM were preserved at both times, and WM was impaired at T1 but recovered during the period of abstinence. These differences among cognitive components may result from differential brain damage or recovery speeds of the cortical areas that are involved. Executive processes, such as inhibition, mental shifting, and WM primarily involve the frontal/prefrontal cortex [48–50]; while simple information storage in STM is associated with more posterior regions and parietal/temporal areas [48–50], and verbal fluency is linked to both frontal and temporal lobes functioning [84]. If neural recovery does occur in remitted AD patients (e.g., [28,85–89], it varies for different brain structures. Van Eijk et al. [28] found a recovery of grey matter volume in regions such as the cingulate gyrus, temporal gyrus, parietal lobule, cerebellum, and precuneus within the first two weeks of abstinence, but not in the precentral gyrus or the frontal gyrus. Our results suggest that posterior regions do not suffer from alcohol abuse to the point that they result in deficits in the associated cognitive functions, while the (pre-)frontal cortex suffers to a greater extent or it recovers more slowly, with probably subtle differences in the exact networks implicated in each function that would explain the different recovery speed. This would have to be tested by brain imaging studies.

Secondly, while our findings are in line with those of several previous studies, they are nonetheless not in agreement with a number of others. Such is the case for our finding that AD exhibit a normal performance in flexibility, verbal fluency, short-term memory, and working memory at some stage during the three weeks of detoxification, whilst other studies have reported deficits for the same functions after longer abstinence periods. This very much reflects the high level of heterogeneity at present regarding data on cognitive impairments and recovery in alcohol abuse. This study was an attempt to focus on more homogeneous groups in terms of the time that has lapsed since the last drink, and it adhered more strictly to assessment points and control factors known to influence recovery. Other factors known to influence the extent of the cognitive impairments seen in AD are the number of previous detoxifications, a family history of alcoholism, age, gender, or smoking. These may have varied significantly between the samples used in previous studies (including ours), and they may explain the observed discrepancies. For example, Moriyama et al. [36] found that the recovery of executive functions was significantly lower in AD who had a prior family history of alcoholism compared to those who did not. It would be relevant to also carefully control for these factors in further studies if a more accurate picture of impairments and their recovery in abstinent AD is to be achieved. The fact that our sample showed an average length of addiction of 9.41 years, which is below the mean duration of alcohol use disorders before first treatment contact (i.e., 18 years [90]) may also explain the absence of deficits in many functions at T1 in this study compared to others.

Thirdly and lastly, the individual analyses have proven to be very informative compared to conventional means comparisons. They have revealed, for example, that even though no significant improvement between the two evaluation points was observed at the group level for inhibition, a considerable proportion of the patients achieved a recovery comparable to that of the control group at T2. They also showed that while on average the patients exhibited a normal performance in WM compared to controls at T2, only half of the patients exhibiting deficits at T1 actually recovered by T2. In light of its benefits in regard to sensitivity, use of this individual analytical procedure is hence recommended for future studies. However, what would be even more informative is to find out the characteristics associated with the different recovery patterns found within the patient groups.

Our study is not without limitations. The reliability of the measures taken on day 1 in patients may be questionable, as this is typically a time of heightened/intense biological and psychological symptoms (e.g., anxiety and withdrawal), which can compromise neurocognitive performance. Ideally, to reflect the effect of alcohol use on cognitive performances before detoxification, the testing should have been performed before the patients initiated detoxification. This could not be done for obvious practical reasons, and the performance would have then depended on the extent of the alcohol intoxication, and it would have varied according to the time of day of the testing. Furthermore, when presenting with a physical dependence, which was the case for the vast majority of the patients tested, they also exhibit anxiety and signs of withdrawal in the morning, before they have their first alcoholic drink which, like benzodiazepines, has a calming effect by altering the GABAergic/Glutamatergic equilibrium. Hence, although imperfect, what alcohol-dependent individuals experience on the first day of abstinence, with benzodiazepine medication, is not completely different from what they experience while actively drinking. Furthermore, the effect of benzodiazepines was controlled for in the analyses. Also, the observed cognitive impairments at T1 cannot be entirely ascribed to the effects of withdrawal as, on average, most of the tasks, and even complex ones such as the Number/Letter task, were executed properly by the patients at T1 while on the other hand, not all of the executive functions recovered (inhibition). All impairments should have disappeared by T2 if the impairment in T1 was only due to the withdrawal-related symptoms. We hence believe that the impairments observed at T1 were mainly due to the effects of a prolonged period of alcohol consumption rather than to withdrawal symptoms.

In conclusion, our results suggest that a 2–3 week alcohol detoxification period is enough to positively impact not only alcohol craving and affective state, but also some higher-level cognitive functions, such as WM, despite the persistence of deficits in others. They also show that individual analysis is of value, as there is variability within the patient groups. An individual evaluation may be of particular use to clinicians as it could allow them to better understand the abilities and deficits their patients may have during and after a 3-week detoxification period, and to implement adapted neurocognitive treatments aimed at reducing the risk of relapse.

### Financial disclosure and competing interest statement

This research was supported by the Belgian National Fund for Scientific Research (FNRS-FRS; grant 3.4585.07). Olivier Luminet and Pierre Maurage are Senior Research Associate at the Belgian National Fund for Scientific Research (FNRS-FRS). The authors have declared that no competing interests exist.
